# State Control of Medical Education

**Published:** 1887-05

**Authors:** 


### *STATE CONTROL OF MEDICAL EDUCATION.*

The medical colleges throughout the United States have generally completed their courses of instruction and a vast number of young men have been turned loose upon the community, armed with the legal right to practice medicine.

There is not a thinking man in the medical profession who does not recognize that the majority of these graduates are insufficiently educated and generally wanting in those high qualities which should be demanded of the candidate for the degree of Doctor of Medicine.

Neither the community nor the profession is benefited by this deluge of poorly-prepared doctors, the only gainers being the medical men who run the colleges as a business investment, but are, at the same time, blind to their duties to that profession which they are supposed to adorn. The best minds in the profession have for years past been outspoken in condemning the iniquities of our present system of medical education, and the JOURNAL has urged with all boldness the application of the only effective remedy, namely, the complete separation of the unfortunate, unwise and debasing co-partnership of the educating and licensing powers. Individual universities like Pennsylvania, Harvard, Yale, Niagara and others may bravely and steadfastly uphold the principles of reform in medical education, but their efforts are unavailing as long as the general law does not interfere to protect the public from the cupidity of those who prostitute their office as teachers for pecuniary advantage. In an editorial on this subject, the *Medical Standard* says:

"There are colleges of deservedly high reputation; others of fair reputation, and still others whose record is the degradation of American medicine and a crime against the American public. The graduates of these schools may be either educated gentlemen, mediocre pretenders, or arrant imposters, yet it has been the custom of most medical practice acts to recognize no distinction, to crush all to a common level, and to endow all with a certificate of competency and authority by the State. It is surprising, in view of this monstrous travesty of law, this enforced degradation of the competent to the legal level of the imposter and quack, this discrimination in effect against schools conducted upon reputable principles, that our educational institutions of acknowledged merit should be relatively so few, and that there should be such a multiplicity of inferior or fraudulent schools? Instead of rewarding the worthy, our system of laws is in effect calculated to discourage and repress them; instead of discountenancing collegiate schemes for personal advertising and personal gain at the sacrifice of professional manhood and the most sacred of obligations to the public, these laws have fostered and welcomed them.

"It is time that medical institutions, conducted primarily for pecuniary profit, were brought to a halt, and the single effective method of doing this is to divest all college diplomas of any legal significance whatever, and lodge all power over registration in the State, represented by a competent examining board."

Four years ago, the JOURNAL advocated the passage of such a law in this State, and by an almost unanimons vote of the Erie County Society, seconded by like action by the majority of the county medical societies of the State, our legislators were asked to pass a bill which would in effect absolutely divorce the educational from the licensing powers. This was, however, strongly opposed by many of the medical colleges and by other influences, backed by the immense wealth of the patent medicine concerns, and failed to become a law. We rejoice, however, to note that the State of Minnesota has taken the lead in this great reform. We publish upon another page the full text of the law which goes into effeet July 1st, and a comparison of its provisions will show them to be almost identical as to effect with the proposed law originating with the Erie County Society in 1883. The principle adopted by the State of Minnesota must, sooner or later, extend to every State in the Union. North Carolina and Virginia have already taken action upon it, and we trust the Empire State may also be among the first in establishing the same safeguard for her citizens. There is not a physician in the United States, (excepting only a few college professors,) who is not personally interested in the furtherance of this movement. The great mass of the medical profession are unable to obtain from their practice an income sufficient to keep their families supplied with the comforts of life, and, at the same time, to keep themselves provided with the latest books and journals and other necessities for the best fulfillment of their responsibilities to their patients; and they always will be poor so long as the supply of doctors *increases three times as fast* as the population of the land.

---

The Virginia State Board of Medical Examiners demand that candidates should attain a standard of seventy-five per cent. in their examinations to obtain a license to practice. Nineteen applicants lately appeared before the Board, and but seven passed satisfactory examinations. Twelve applicants were rejected. Each of the candidates was fresh from college with his diploma.

---

The Nashville *Medical News*, the *Medical Standard* of Chicago, the *Southwestern Medical Gazette*, Louisville,—

"Spring hangs her infant blossoms on the trees,
Rocked in the cradle of the western breeze;"

and the latest blossoms on the old tree of medical journalism are the above-named journals. They are all heartily welcomed to the list of our exchanges, as they give evidence of editorial ability and earnest purpose to advance the best interests of the profession.

We desire to correct a very plain mistake in the heading of the article published in the April number, from the pen of the senior editor of this Journal. The subject is "Intra-Uterine and Vaginal *Irrigation*," instead of *Irritation*. Our proof-reader is chargeable with this error. In the hurry of getting out a monthly journal, mistakes will happen and our readers will overlook them.

---

The death of Dr. Wm. Ring, of this city, which occurred last week, was keenly felt by all the profession. The Erie County Medical Society met to express their sorrow at his so sudden death. A full report of the meeting, as well as a history of the doctor, will appear in our next issue.

